# Artificial Intelligence in the Diagnosis and Management of Atrial Fibrillation

**DOI:** 10.3390/diagnostics15202561

**Published:** 2025-10-11

**Authors:** Otilia Țica, Asgher Champsi, Jinming Duan, Ovidiu Țica

**Affiliations:** 1 Cardiology Clinic, Emergency County Clinical Hospital of Bihor, 410165 Oradea, Romania; 2 Cardiovascular Sciences, College of Medicine and Health, University of Birmingham, Birmingham B15 2TT, UK; a.champsi@bham.ac.uk; 3 Division of Informatics, Imaging and Data Sciences, University of Manchester, Manchester M13 9PL, UK; j.duan@bham.ac.uk; 4 School of Computer Science, University of Birmingham, Birmingham B15 2TT, UK; 5 Department of Morphological Disciplines, Faculty of Medicine and Pharmacy, University of Oradea, 410073 Oradea, Romania; 6 Pathology Department, Emergency County Clinical Hospital of Bihor, 410165 Oradea, Romania

**Keywords:** artificial intelligence, atrial fibrillation, deep learning, machine learning, convolutional neural networks, wearable devices, risk prediction

## Abstract

Artificial intelligence (AI) has increasingly become a transformative tool in cardiology, particularly in diagnosing and managing atrial fibrillation (AF), the most prevalent cardiac arrhythmia. This review aims to critically assess and synthesize current AI methodologies and their clinical relevance in AF diagnosis, risk prediction, and therapeutic guidance. It systematically evaluates recent advancements in AI methodologies, including machine learning, deep learning, and natural language processing, for AF detection, risk stratification, and therapeutic decision-making. AI-driven tools have demonstrated superior accuracy and efficiency in interpreting electrocardiograms (ECGs), continuous monitoring via wearable devices, and predicting AF onset and progression compared to traditional clinical approaches. Deep learning algorithms, notably convolutional neural networks (CNNs) and recurrent neural networks (RNNs), have revolutionized ECG analysis, identifying subtle waveform features predictive of AF development. Additionally, AI models significantly enhance clinical decision-making by personalizing anticoagulation therapy, optimizing rhythm versus rate-control strategies, and predicting procedural outcomes for catheter ablation. Despite considerable potential, practical adoption of AI in clinical practice is constrained by challenges including data privacy, explainability, and integration into clinical workflows. Addressing these challenges through robust validation studies, transparent algorithm development, and interdisciplinary collaborations will be crucial. In conclusion, AI represents a paradigm shift in AF management, promising improvements in diagnostic precision, personalized care, and patient outcomes. This review highlights the growing clinical importance of AI in AF care and provides a consolidated perspective on current applications, limitations, and future directions.

## 1. Introduction

Atrial fibrillation (AF) is the most common sustained cardiac arrhythmia, characterized by irregular and often rapid heart rhythms resulting from disorganized atrial electrical activity. Globally, AF affects approximately 33 million people [1], with rising prevalence attributed to aging populations, increasing prevalence of cardiovascular risk factors such as hypertension and diabetes, and better survival rates from cardiac diseases [2,3,4,5]. Early and accurate diagnosis of AF is clinically crucial, significantly decreasing the risk of adverse outcomes, including stroke [6], heart failure [7], and cardiovascular mortality [8], through timely intervention, effective anticoagulation management, and personalized therapy strategies [9,10].

Artificial intelligence (AI) has rapidly emerged as a revolutionary force within healthcare, particularly cardiology, offering transformative capabilities in disease prediction, diagnostics, personalized treatment, and clinical decision-making. Recent landmark studies have highlighted AI’s potential, particularly in improving clinical outcomes and resource optimization through precise disease prediction and personalized interventions [11,12,13]. AI methodologies such as machine learning, deep learning, and natural language processing are being increasingly utilized to enhance diagnostic precision, improve patient outcomes, and optimize resource allocation [14]. Specifically, in cardiology, AI systems have shown substantial potential to surpass traditional diagnostic methods, particularly in interpreting electrocardiograms (ECGs) and managing complex data from wearable devices and electronic health records. The integration of AI into clinical practice promises substantial improvements in diagnosing AF earlier and more accurately, thereby reducing morbidity and enhancing patient-centered care [15]. The objective of this review is to provide a comprehensive overview of state-of-the-art AI technologies applied to AF management, identify their clinical benefits and limitations, and explore their potential to reshape diagnostic and therapeutic strategies. By focusing on diagnostic tools, predictive models, and decision-support systems, this article aims to inform clinicians, researchers, and developers on the current landscape and future opportunities of AI in atrial fibrillation care.

Importantly, this review is designed to inform clinicians and healthcare professionals about the clinical applications of AI in AF, rather than to provide a technical dissection of machine learning models. Our goal is to bridge the gap between data science and patient care by presenting AI advancements in a clinically meaningful and accessible manner.

This review makes a distinct contribution to the literature by bridging computational AI methodologies with their direct clinical implementation in atrial fibrillation (AF) care. Unlike prior reviews that typically focus on either technical algorithm development or clinical practice in isolation, our work integrates both perspectives. In particular, we emphasize clinically actionable AI strategies, the integration of wearable and implantable monitoring technologies, and the ethical and regulatory considerations that influence translation into practice. By combining these dimensions, this review provides a consolidated perspective that supports both researchers and clinicians in advancing AI-driven AF management.

The remainder of this review is organized as follows: Section 2 outlines the methods used in conducting this narrative review and summarizes core AI methodologies and datasets relevant to AF. Section 3 discusses AI applications in AF diagnosis, including ECG interpretation, wearable and implantable technologies, and comparative studies against traditional diagnostic methods. Section 4 examines AI-based risk prediction and stratification models, integrating clinical, genomic, and big data approaches. Section 5 explores AI applications in therapeutic decision-making, including anticoagulation management, rhythm-control strategies, heart rate variability, and catheter ablation planning. Section 6 highlights key challenges and limitations, such as data quality, bias, explainability, and regulatory barriers. Section 7 looks ahead to future directions and emerging methodologies, while Section 8 presents the conclusions and summarizes the unique contributions of this review.

## 2. Methods and Approaches in AI for AF

Given the heterogeneity and rapid evolution of the field, we conducted a narrative review rather than a systematic review, aiming to synthesize key clinical and computational advances in AI for atrial fibrillation while acknowledging the inherent methodological limitations of this approach.

To identify relevant literature, we searched PubMed, Scopus, and Web of Science for peer-reviewed articles published between January 2018 and March 2025. The search strategy used combinations of the following keywords: “artificial intelligence,” “atrial fibrillation,” “deep learning,” “machine learning,” “electrocardiogram,” “wearable devices,” “risk prediction,” and “therapeutic decision-making.” We included only English-language studies that reported clinical, translational, or methodological insights directly relevant to AF diagnosis, risk stratification, or management. Exclusion criteria were non-peer-reviewed sources, conference abstracts without full manuscripts, and studies not directly related to AF. Given the narrative nature of this review, we did not apply formal systematic review protocols; thus, potential limitations include selection bias and incomplete coverage. To mitigate these, we prioritized high-impact journals and landmark validation studies to ensure both breadth and clinical relevance.

This review is presented as a narrative review rather than a systematic review. While this approach allows for a broad synthesis of recent findings across computational and clinical domains, it does not follow formal systematic review protocols. As such, potential limitations include selection bias and incomplete coverage of all available literature. To mitigate these, we prioritized high-impact, peer-reviewed studies published between 2018 and 2025, and we highlight landmark validation studies where available.

To strengthen the methodological foundation of this review and clarify the computational strategies used in atrial fibrillation (AF) analysis, we include a concise overview of core artificial intelligence (AI) techniques commonly employed in this field. Convolutional neural networks (CNNs) are widely used for spatial feature extraction from ECG waveforms, while recurrent neural networks (RNNs), including Long Short-Term Memory (LSTM) models, are employed to model temporal dependencies in sequential cardiac signals. Transformer-based architectures, which utilize attention mechanisms to capture long-range relationships in time-series data, represent a promising frontier in AF modeling. In evaluating model performance, key metrics such as area under the receiver operating characteristic curve (AUC), F1 score, sensitivity, specificity, and positive predictive value (PPV) are essential for robust validation. Furthermore, we outline common validation strategies—such as k-fold cross-validation, hold-out testing, and prospective cohort analysis—that help ensure model generalizability [16]. Optimization methods including regularization, dropout, batch normalization, and learning rate scheduling are also highlighted as essential tools for enhancing performance and preventing overfitting. This technical synthesis complements the clinical emphasis of this review by bridging algorithmic foundations with practical implementation considerations.

### 2.1. Overview of Machine Learning and Deep Learning Techniques

Machine learning (ML) and deep learning (DL) represent two principal branches of AI extensively employed in the study and management of AF. Machine learning algorithms, including logistic regression, decision trees, random forests, and support vector machines, analyze large datasets to detect patterns and make predictions based on statistical modeling [17,18]. In contrast, deep learning, a subset of ML inspired by neural network structures [19] leverages multiple processing layers to identify complex data patterns automatically [12].

### 2.2. Explanation of CNNs, RNNs, Transformers, and Other AI Techniques

**Figure 1:**
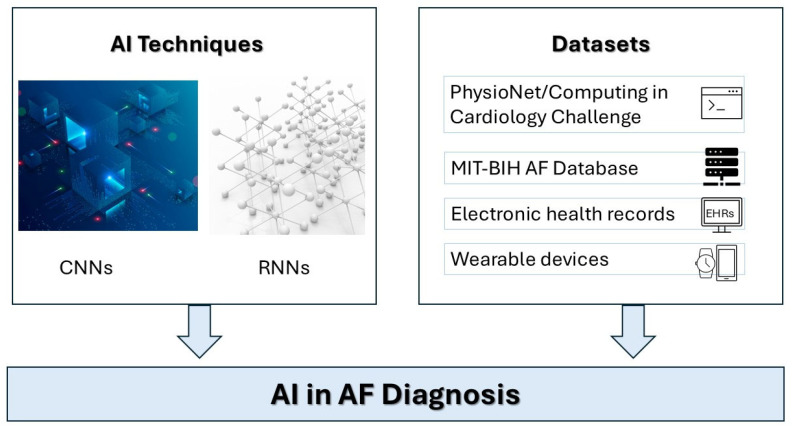
Architectures of Deep Learning Models for ECG Analysis Illustration of convolutional neural networks (CNNs) for spatial feature extraction and recurrent neural networks (RNNs) for temporal pattern recognition in atrial fibrillation diagnosis. Emerging circulating biomarkers in heart failure, AF, atrial fibrillation; AI, artificial intelligence; CNNs, convolutional neural networks; RNN, recurrent neural networks.

Deep learning techniques frequently utilized in AF include convolutional neural networks (CNNs) and recurrent neural networks (RNNs), as summarized in Figure 1. CNNs specialize in spatial hierarchical feature extraction, making them ideal for interpreting imaging data such as ECGs. CNNs excel at identifying subtle morphological changes in ECG waveforms that predict AF episodes with high accuracy [13]. On the other hand, RNNs, including variants such as Long Short-Term Memory (LSTM) networks, are specifically designed to analyze sequential data, capturing temporal dynamics inherent in continuous ECG recordings or heart rate data collected from wearable devices [12,20,21].

In addition to established architecture, recent AI research has embraced transformer-based models that utilize attention mechanisms to capture long-range temporal dependencies in sequential data such as ECG signals [22]. Self-supervised learning approaches, which enable models to extract meaningful representations from unlabeled data, are particularly valuable in real-world clinical datasets where annotated data may be limited. Federated learning frameworks, meanwhile, support decentralized model training across multiple institutions without compromising patient data privacy—an increasingly critical consideration in the development of AI for AF care [23,24].

Self-supervised learning offers a powerful approach for leveraging large, unlabeled ECG datasets—especially valuable in healthcare, where annotation is resource-intensive. Models that learn underlying signal representations from raw data without supervision can enhance robustness and adaptability in AF detection, particularly in underrepresented populations or rare arrhythmia phenotypes.

Transformers have solidified their position as the state-of-the-art architecture in natural language processing (NLP) and beyond, driven by their scalability, parallelism, and ability to capture complex patterns in data. Recent models such as GPT-4 [25], PaLM 2 [26], and LLaMA 2 demonstrate how Transformer-based architectures continue to push the boundaries of language understanding and generation. These models outperform earlier benchmarks across a wide range of tasks, including reasoning, summarization, and multilingual processing, highlighting the enduring dominance of Transformers in AI research and applications.

### 2.3. Data Sources and Common Datasets Used in AF Studies

Key datasets in AF studies typically include large ECG repositories such as the PhysioNet/Computing in Cardiology (CinC) Challenge dataset, MIT-BIH Atrial Fibrillation Database, and extensive patient electronic health records (EHRs) [27,28]. Additionally, wearable technology-derived data [29], such as those from Apple Watch, Fitbit, and other continuous heart-monitoring devices, are increasingly integrated to enhance the predictive accuracy and diagnostic capabilities of AI systems. Collectively, these datasets enable robust model training, validation, and clinical applicability of AI methodologies in AF management [30,31].

## 3. AI in AF Diagnosis

This section provides an overview of how artificial intelligence has been applied to improve atrial fibrillation (AF) diagnosis. AI plays two complementary roles in this domain. First, it enhances the accuracy of automatic rhythm recognition from wearable and implantable devices, enabling early detection and large-scale community screening. Second, it supports clinician-led diagnosis by improving the precision, consistency, and efficiency of ECG interpretation in clinical practice. Together, these applications illustrate how AI strengthens both patient-driven monitoring and physician-based diagnostic decision-making.

### 3.1. AI-Driven ECG Analysis Techniques

Recent developments in mobile health have enabled AI to extend its diagnostic capabilities into non-traditional clinical settings, such as homes and communities. Wearable technologies, coupled with AI-based signal interpretation, now support large-scale, passive rhythm monitoring, allowing earlier detection of AF in asymptomatic individuals and populations at risk. These innovations represent a shift toward more accessible, patient-centered AF care [32].

AI-driven ECG analysis has significantly advanced AF diagnosis by enabling precise, automated detection of AF episodes. Algorithms leveraging CNNs have demonstrated remarkable accuracy in identifying AF from standard 12-lead and single-lead ECGs. These algorithms automatically analyze waveform morphology, interval variability, and subtle irregularities that traditional diagnostic methods might overlook, thus achieving superior sensitivity and specificity [16,33,34].

### 3.2. Wearable and Implantable Technologies

**Figure 2:**
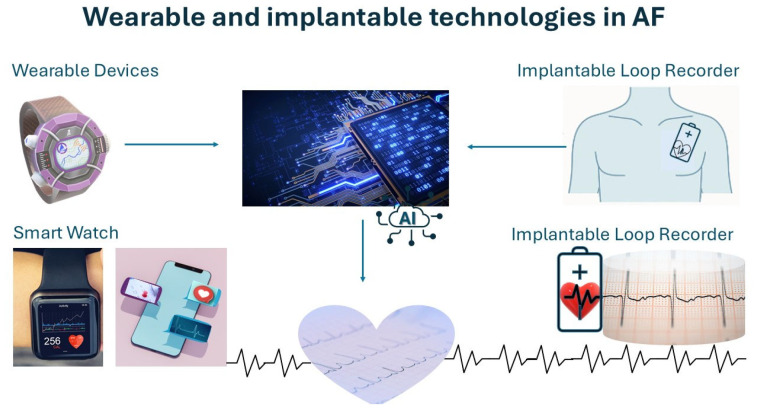
Wearable and Implantable Technologies for AF Detection. Visual representation of smartwatches, fitness trackers, and implantable loop recorders integrated with AI for continuous rhythm monitoring and early AF detection. Liquid biopsy and non-invasive molecular tools in cardiovascular disease.

Wearable technologies have further revolutionized AF diagnostics by facilitating continuous cardiac monitoring in real-time outside clinical settings, as seen in Figure 2. Devices such as the Apple Watch, Fitbit, and implantable loop recorders offer extensive longitudinal data collection capabilities. These technologies, integrated with AI models, detect irregular rhythms earlier and alert users promptly, enabling timely clinical interventions and reducing stroke risk significantly [29,35].

Recent studies have also demonstrated the feasibility and effectiveness of fully digital, self-screening approaches using patch-based ECG devices, which enhance patient autonomy while maintaining diagnostic accuracy. For example, a recent study [36] evaluated a fully digital self-screening model using patch ECGs and confirmed its potential as a scalable strategy for AF detection in community settings. Such advances in patient-centered, biointegrated monitoring solutions will play a pivotal role in expanding AI-driven cardiac diagnostics beyond traditional clinical environments.

Recent advances in biointegrated and optoelectronic cardiac monitoring devices offer new possibilities for continuous, real-time AF detection. These include flexible, skin-conformal sensors and implantable optoelectronic systems that provide high-fidelity data while maintaining patient comfort [37,38]. Integrating AI with such platforms could improve early AF detection and enable scalable, minimally invasive diagnostics beyond conventional ECG monitoring.

### 3.3. Comparative Studies Versus Traditional Diagnostic Methods

Comparative studies underscore AI’s superiority in diagnostic performance compared to traditional methods, as seen in Table 1. For instance, CNN-based AI systems have consistently outperformed cardiologists and standard software algorithms in accuracy and speed when analyzing large-scale ECG databases, demonstrating a promising future for AI-enhanced AF management [39]. It is important to note, however, that while large-scale database analyses demonstrate scalability and efficiency, the gold standard for evaluating diagnostic accuracy at the patient level remains physician-confirmed ECG interpretation or reference ECG patch monitoring. Accordingly, AI model performance is typically benchmarked against these clinical standards, ensuring that diagnostic accuracy is validated in the context most relevant to individual patient care.

These studies, many of which have appeared in high-impact journals, demonstrate the superior diagnostic accuracy, predictive value, and efficiency of AI tools, supporting their emerging role in clinical AF management. In this context, it is important to highlight how landmark contributions have shaped the current state-of-the-art. In recent years, several landmark studies published in high-impact journals have significantly advanced the field of artificial intelligence in atrial fibrillation (AF) care. These studies have demonstrated the clinical utility of deep learning models for early AF detection from sinus rhythm ECGs, large-scale wearable device monitoring, and individualized risk prediction using multimodal data sources. For instance, Attia et al. and Raghunath et al. have shown that AI can detect latent AF from standard ECGs with high accuracy, while large population studies like the Apple Heart Study and the Fitbit Heart Study have validated AI-powered wearables in community screening. Incorporating and critically evaluating these seminal works strengthens the evidence base of this review, situates its content within the current state-of-the-art, and underscores the transformative potential of AI technologies in reshaping AF diagnosis and management.

To illustrate the clinical utility of artificial intelligence in AF, Table 1 has been divided into two subsections to reflect distinct clinical tasks. The first subsection summarizes studies that evaluated the diagnostic accuracy of AI in detecting AF compared to cardiologists or standard software. The second subsection highlights predictive studies, where AI models analyze sinus rhythm ECGs to forecast the future development of AF. Separating these categories clarifies the unique role and implications of AI in both immediate diagnosis and long-term risk prediction. Studies were selected based on their relevance, methodological rigor, and contribution to the advancement of AI-driven ECG interpretation, wearable technology [40], and risk stratification in AF. These representative examples highlight the performance, strengths, and clinical applicability of various AI approaches in real-world and research settings.

In addition to the studies summarized in Table 1, large-scale community trials such as the Apple Heart Study [41] and the Fitbit Heart Study [40] have validated AI-powered wearable devices for AF detection at scale, underscoring their feasibility for population-level screening. Likewise, Attia et al. (2019) [42] demonstrated that AI models could predict latent AF during sinus rhythm from standard 12-lead ECGs, supporting earlier identification of at-risk individuals. Incorporating these landmark studies strengthens the clinical evidence base and highlights the translation of AI technologies from research into real-world practice.

Molecular and pathophysiological pathways—such as TGF-β1 signaling—play a key role in atrial remodeling and fibrosis, offering potential targets for AI-guided therapeutic strategies in atrial fibrillation [43]. AI-guided AF management can be enriched by incorporating mechanistic insights from cardiac pathophysiology. For instance, TGF-β1 signaling pathways involved in atrial fibrosis represent actionable biomarkers for stratifying patients [44,45]. Interventional studies—such as those investigating agents like Xanthohumol—should be linked with AI frameworks to personalize treatment selection and therapeutic planning.

**Table 1:** Comparative studies demonstrating AI superiority over traditional diagnostic methods.

Study (Year)	AI Method	Traditional Comparator	Performance	Key Findings	Reference
Diagnostic accuracy studies of AI in AF detection					
Hannun et al. (2019)	CNN analyzing 12-lead & single-lead ECGs	Board-certified cardiologists	Sensitivity 98.0%, Specificity 99.0% vs. Cardiologists 90.0%/93.0%	CNN achieved cardiologist-level arrhythmia classification, outperforming human experts.	[33]
Ribeiro et al. (2020)	DNN trained on >2 M ECGs	Cardiology residents	F1 > 80%, Specificity >99%	DNN outperformed residents across multiple arrhythmia classes.	[46]
Poh et al. (2018)	DCNN on PPG waveforms	Cardiologist-reviewed ECG	Sensitivity 95–100%, Specificity 99%	PPG-based deep learning achieved rapid and accurate AF detection.	[47]
Tison et al. (2018)	DNN on smartwatch PPG	Reference 12-lead ECG	Sensitivity 98%, Specificity 90%	Demonstrated feasibility of passive AF detection, though performance declined in ambulatory settings.	[48]
Apple Heart Study (2019)	PPG-based irregular pulse algorithm (wearable)	ECG patch monitoring	PPV 84%	Validated large-scale, site-less AF screening using wearables.	[41]
Fitbit Heart Study (2022)	PPG irregular rhythm detection	ECG patch monitoring	PPV 98.2% (concurrent detection)	Consumer wearables matched traditional ECG detection accuracy.	[40]
AliveCor KardiaMobile (2023)	AI algorithm on single-lead handheld ECG	Cardiologist interpretation	Sensitivity 98.5%, Specificity 91.4%	Enabled accurate, portable AF detection in telemedicine contexts.	[49]
Vasconcelos et al. (2023)	FD-CNN with transfer learning	Standard ECG classification	High accuracy across centers	Robust multicenter ECG classification with strong generalizability.	[24]
Gill et al. (2024)	Wearable monitoring in RATE-AF trial	Standard HR evaluation (digoxin vs. β-blockers)	Comparable to standard methods	Demonstrated feasibility of consumer wearables for AF management.	[29]
Johnson et al. (2025)	AI-enabled physician-ready ECG reports	Cardiologists	Comparable/superior accuracy	Validated automated AI reporting for real-world ECGs.	[13]
Predictive studies of AF development during sinus rhythm					
Attia et al. (2019)	DNN on standard 12-lead ECG	Standard ECG interpretation	AUC 0.87 vs. 0.79	Predicted AF during sinus rhythm with higher accuracy.	[42]
Raghunath et al. (2021)	DNN on 12-lead ECG	CHARGE-AF clinical risk score	AUC 0.87 vs. 0.78	More accurate prediction of new-onset AF than CHARGE-AF.	[50]
AI-ECG Early AF Detection (2024)	DNN on 12-lead ECG	Standard ECG	Sensitivity 90%, Specificity 80%	Improved detection of paroxysmal AF during routine screening.	[51]
Cho et al. (2025)	AI-derived ECG “biological age” models	Clinical risk factors	Strong correlation with AF risk	Multinational study validating ECG age as a predictor of AF.	[39]

Abbreviations: AF, Atrial Fibrillation; AUC, Area Under the Receiver Operating Characteristic Curve; CHARGE-AF, Cohorts for Heart and Aging Research in Genomic Epidemiology Atrial Fibrillation Risk Score; CI, Confidence Interval; CNN, Convolutional Neural Network; DCNN, Deep Convolutional Neural Network; DNN, Deep Neural Network; ECG, Electrocardiogram; F1, F1 Score (harmonic mean of precision and recall); IHRD, Irregular Heart Rhythm Detection; PPG, Photoplethysmography; PPV, Positive Predictive Value.

The table provides a summary of selected comparative studies evaluating the performance of artificial intelligence (AI) methods versus traditional diagnostic approaches in atrial fibrillation (AF). Each entry includes the study year, AI methodology used (e.g., CNN, DNN), the traditional comparator, performance metrics (e.g., sensitivity, specificity, AUC), and key findings. These studies demonstrate the superior diagnostic accuracy, predictive value, or efficiency of AI tools, supporting their emerging role in clinical AF management.

## 4. Risk Stratification and Prediction of AF

This section explores the use of AI for predicting the incidence, progression, and clinical outcomes of atrial fibrillation. We review predictive modeling approaches based on clinical, genomic, and biomarker data, as well as the role of big data analytics and multimodal integration. Together, these studies illustrate how AI enhances risk stratification and supports earlier, more tailored interventions for patients at risk of AF.

### 4.1. AI-Based Prediction Models for AF Incidence and Progression

**Figure 3:**
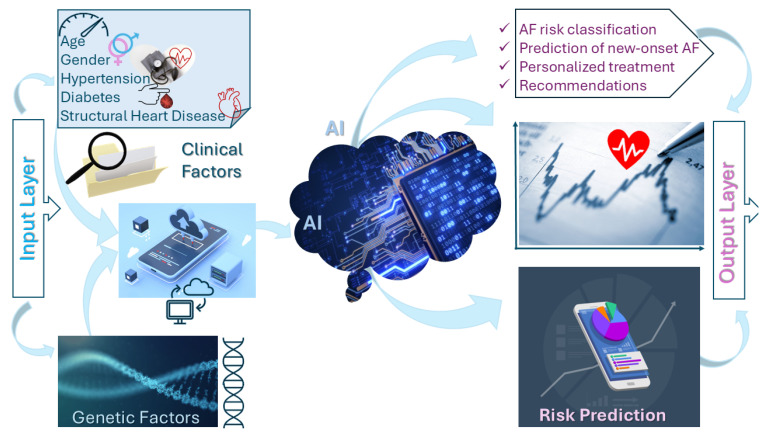
AI-Based Risk Stratification Using Clinical and Genetic Data. Schematic diagram of AI models integrating electronic health records (EHRs), genomic variants, and patient-specific factors to predict AF risk and guide clinical decision-making. AI applications in heart failure diagnostics. AI, artificial intelligence.

Artificial intelligence (AI) has dramatically enhanced the capability to predict atrial fibrillation (AF) incidence and progression, significantly surpassing traditional clinical risk stratification models as shown in Figure 3. AI-driven predictive models integrate extensive clinical data, patient demographics, ECG characteristics, and advanced biomarkers [52] to accurately forecast AF development and its clinical trajectory. These models utilize techniques such as logistic regression, random forests, and deep learning approaches, particularly convolutional neural networks (CNNs) and recurrent neural networks (RNNs), to handle complex and multidimensional data [53,54,55].

Several predictive algorithms have recently demonstrated the potential of AI to outperform traditional clinical models. For example, an AI-ECG model that could predict atrial fibrillation from sinus rhythm recordings with an AUC of 0.87, significantly higher than conventional ECG interpretation (AUC 0.79), was developed [42]. Similarly, it was reported [50] that a deep neural network trained on standard 12-lead ECGs predicted new-onset AF more accurately than the widely used CHARGE-AF clinical risk score (AUC 0.87 vs. 0.78). These findings highlight the ability of AI to identify subtle electrocardiographic signatures invisible to the human eye, thereby enabling earlier identification of at-risk individuals.

Beyond ECG-based models, AI has also been integrated with polygenic risk scores and biomarker data to refine risk stratification. Recent work has demonstrated that combining genetic variants with AI-driven ECG features enhances predictive performance compared to genetics or clinical scores alone. In 2025, Kim et al. [56] proposed a machine learning-based plasma protein risk score that improved AF prediction compared with established clinical and genomic models. Similarly, an “AI-derived ECG age” validated metric [39] across multinational cohorts, confirming its association with future AF risk beyond traditional risk factors.

Collectively, these examples illustrate that AI-based predictive algorithms not only surpass existing tools such as CHARGE-AF but also open new avenues by incorporating multimodal data sources, including genomics, proteomics, and imaging. Such approaches signal a shift toward precision risk prediction in AF, which—pending prospective validation—could enable earlier and more personalized preventive interventions.

Modern AI approaches also incorporate polygenic risk scores [57] and circulating biomarkers [52], enhancing the precision of predictive models [56,58]. This integration of multi-omics data [59] enables more refined stratification of AF risk [50] and facilitates earlier, tailored interventions.

#### Prospective Validation of AI Predictive Models

A critical step in establishing the clinical utility of predictive AI models is their prospective validation in independent cohorts. Several notable trials have addressed this requirement. The Apple Heart Study [41] and the Fitbit Heart Study [40] provided large-scale, prospective evidence supporting the feasibility of AI-enabled wearables for AF detection and population-level screening. Importantly, these trials demonstrated not only technical accuracy but also the scalability of AI approaches in real-world community settings.

In addition, Noseworthy et al. conducted a prospective interventional trial showing that an AI-ECG model could predict latent AF during sinus rhythm and successfully guide targeted screening strategies. These findings validated earlier retrospective results and underscored the potential for AI to identify patients who might otherwise remain undiagnosed until complications arise.

Ongoing studies such as the REGAL trial are now testing AI-ECG algorithms in pragmatic, decentralized, randomized controlled settings. These trials are expected to provide further evidence on clinical effectiveness, cost-effectiveness, and workflow integration in primary care. Collectively, these prospective validations demonstrate that AI models can perform robustly outside retrospective datasets and highlight the need for continued large-scale clinical trials to secure regulatory and clinical acceptance.

### 4.2. Integration of Big Data Analytics

Big data analytics has further augmented the potential of AI in AF risk stratification and prediction. Electronic health records (EHRs) [60], large-scale genetic databases, wearable device datasets, and longitudinal observational data constitute a vast resource for developing robust predictive algorithms. The integration of these diverse data sources into AI algorithms facilitates the identification of subtle and previously unrecognized patterns predictive of AF. For example, the use of big data analytics and machine learning algorithms applied to EHRs and genomic datasets has resulted in significant improvements in AF prediction and individualized patient risk profiles [30,53,61].

Despite these advances, ongoing challenges remain, including ensuring data quality, managing biases inherent in large datasets, and addressing ethical considerations regarding data privacy. Furthermore, standardized approaches and interoperability between different data sources and healthcare systems are required for broad clinical adoption. Continued refinement and validation of AI algorithms, combined with multidisciplinary collaborations and robust regulatory frameworks, are essential for translating these predictive tools into routine clinical practice.

## 5. AI in Therapeutic Decision-Making

This section focuses on how AI informs and optimizes therapeutic strategies for atrial fibrillation. We review applications in personalizing anticoagulation and stroke prevention, predicting responses to rhythm-control interventions, integrating heart rate variability into monitoring frameworks, and improving procedural planning and outcome prediction for catheter ablation. These advances highlight the growing role of AI in guiding precision medicine approaches in AF management.

Recent studies provide concrete illustrations of how AI is being applied to guide treatment decisions in atrial fibrillation. For example, AI-based anticoagulation decision-support systems have been developed to integrate CHA_2_DS_2_-VA risk scores with additional patient-specific factors such as bleeding risk, comorbidities, and genetic profiles, thereby offering more individualized anticoagulation recommendations [62]. In rhythm-control strategies, machine learning models trained on clinical histories, ECG features, and imaging data have been used to predict which patients are most likely to benefit from antiarrhythmic drugs or catheter ablation [63]. In procedural contexts, deep learning models applied to anatomical imaging and electrogram data have demonstrated value in identifying ablation targets and predicting recurrence risk. A recent study [64] reported that CNN-based models could accurately localize trigger origins during ablation, while Tabaja et al. [65] introduced electroporation-guided AI models that improved procedural planning and outcome prediction. These examples highlight the movement of AI from theoretical promise to practical tools that can directly inform therapy selection and optimize patient outcomes.

### 5.1. Personalizing Anticoagulation and Stroke Prevention

AI has become instrumental in personalizing therapeutic approaches to atrial fibrillation (AF), particularly in anticoagulation management and stroke prevention. Traditional anticoagulation therapy relies on standardized risk stratification scores like CHA2DS2-VA. However, AI algorithms integrate broader clinical data, genetic markers, patient-specific bleeding risks, and lifestyle factors to tailor anticoagulant choices and dosages precisely. Recent studies demonstrate that AI-driven anticoagulation management significantly reduces adverse events such as stroke and bleeding complications compared to conventional risk scores [2,62].

### 5.2. Predicting Responses to Rhythm-Control Strategies

In rhythm-control strategies, AI provides valuable predictive insights regarding patient responsiveness to medication and procedural interventions. Machine learning models utilizing clinical history, ECG patterns, imaging data, and biomarkers predict the likelihood of successful restoration and maintenance of sinus rhythm, allowing clinicians to choose the most effective rhythm-control strategies proactively. Deep learning models, especially those employing CNNs and RNNs, have shown excellent predictive accuracy, surpassing traditional clinical decision-making methods [63,66].

### 5.3. Heart Rate Variability and AI-Based Monitoring

Heart rate variability (HRV) is a well-established biomarker of autonomic modulation and arrhythmia risk. Integrating HRV features with AI models can enhance early AF detection and disease progression monitoring. We will incorporate recent studies on HRV analytics and arrhythmia dynamics, linking them to machine learning-based prediction and monitoring frameworks in AF care [67].

### 5.4. AI for Procedural Planning and Outcome Prediction in Catheter Ablation

AI also significantly enhances procedural planning and outcome prediction for catheter ablation, a common invasive treatment for AF. Predictive models developed using large databases from previous ablation outcomes, patient-specific anatomical imaging, and real-time procedural data have improved the precision of ablation strategies. AI-driven systems can accurately identify critical anatomical targets, optimize procedural parameters, and predict ablation success and recurrence rates with high accuracy. Such predictive analytics support clinicians in selecting suitable candidates for ablation, optimizing procedural techniques, and improving patient counseling [64,65].

These advances underscore the importance of AI in transitioning towards precision medicine in AF management, enabling more targeted and effective patient care [13,68]. However, practical implementation in clinical practice faces notable challenges. Key among these are the transparency and interpretability of AI algorithms, which are critical for gaining clinician trust and ensuring accountability [12]. Additionally, the standardized integration of AI tools into existing clinical workflows remains complex, necessitating comprehensive training, infrastructure adjustments, and clear regulatory frameworks [11,69].

Recent work has highlighted the importance of transparent and clinically applicable machine learning models in atrial fibrillation care [58]. A recent study developed an explainable machine learning framework for outcome prediction in patients with non-valvular AF using data from the large-scale GLORIA-AF registry [70]. This study illustrates how interpretable AI tools can be used to support risk stratification and clinical decision-making, reinforcing the need for similar explainability and validation standards in AI applications for AF diagnosis and management. Continuous validation of these AI tools through robust prospective clinical trials is essential to establish their reliability, efficacy, and safety before widespread adoption [22,71]. Furthermore, addressing ethical considerations, particularly concerning data privacy, patient autonomy, and potential biases in algorithmic decisions, is crucial to ensure equitable and ethical implementation of AI solutions [72]. Finally, fostering interdisciplinary collaborations between clinicians, data scientists, ethicists, and regulatory bodies will significantly enhance the practical and effective deployment of AI in therapeutic decision-making for AF management [50,72].

## 6. Challenges and Limitations

### 6.1. Data Quality, Heterogeneity, and Biases

A major limitation of AI in atrial fibrillation (AF) care is the dependence on large, high-quality datasets. In practice, clinical data often contain noise, missing values, and variability in acquisition methods, which may reduce diagnostic accuracy and reproducibility. Differences in ECG fidelity across wearable devices and recording systems can also introduce inconsistencies, limiting cross-platform generalizability.

Bias embedded in training datasets represents an equally important challenge. Demographic, geographic, and socioeconomic underrepresentation can lead to algorithms that perform suboptimally in certain populations, exacerbating healthcare disparities. To address these issues, future AI development must prioritize inclusive, multicenter datasets and apply fairness-aware strategies such as demographic stratification, subgroup performance auditing, and bias-mitigation techniques (e.g., reweighting, adversarial debiasing). Such approaches are essential to ensure equitable performance and clinical reliability across diverse patient populations [73,74,75,76,77,78].

Bias in training datasets represents a major limitation for AI in AF care. Demographic and socioeconomic underrepresentation may result in algorithms that perform poorly in specific populations, potentially widening existing healthcare disparities. To address this, fairness-aware approaches, subgroup performance audits, and multicenter validation across diverse cohorts are essential. In addition, the opacity of deep learning “black box” models limits interpretability and hinders clinician trust. Explainable AI techniques (e.g., SHAP, LIME, Grad-CAM) should be integrated into AF diagnostic and therapeutic platforms to provide transparency and actionable rationale. Finally, regulatory barriers remain a significant challenge, as standards for validation, approval, and liability frameworks are still evolving. Close collaboration with regulatory agencies will be necessary to establish clinically acceptable guidelines for AI integration.

### 6.2. Ethical and Privacy Concerns

The integration of AI into AF care requires continuous monitoring and the collection of sensitive health data, raising concerns about patient consent, data ownership, and security. Compliance with regulations such as the European GDPR and the U.S. HIPAA is essential, but practical implementation remains challenging in real-world settings [79,80].

To safeguard privacy, approaches such as federated learning and differential privacy have been introduced, allowing models to be trained collaboratively without directly sharing patient-level data. Robust anonymization, secure data governance, and encryption strategies are equally important to maintain patient trust. As AI systems move closer to routine clinical use, embedding these privacy-preserving technologies within scalable, regulatory-compliant frameworks will be critical to ensure both ethical integrity and public acceptance [81,82,83,84].

### 6.3. Regulatory Barrier and Clinical Acceptance

Beyond data quality and privacy, regulatory approval and clinician acceptance remain major barriers to AI integration in AF care. The “black box” nature of many deep learning models limits interpretability, reduces clinician trust, and complicates approval by agencies such as the FDA and EMA. Clearer standards for algorithm validation, liability frameworks, and post-deployment monitoring are urgently needed to ensure safe and accountable implementation [13,69]. Improving clinical acceptance will require explainable AI (XAI) approaches—such as SHAP, LIME, or attention heatmaps—that provide intuitive rationales for model outputs. Equally important are user-friendly interfaces embedded into existing clinical workflows, supported by clinician training. Achieving regulatory endorsement and real-world usability will therefore depend on balancing transparency, safety, and practicality, ensuring that AI tools enhance rather than disrupt everyday patient care [58].

## 7. Future Directions

### 7.1. Emerging AI Technologies and Methodologies

The future of artificial intelligence (AI) in atrial fibrillation (AF) care will be shaped by the emergence of novel technologies, greater integration into clinical settings, and interdisciplinary collaboration. Cutting-edge AI methodologies, such as federated learning, self-supervised learning, and transformer-based models, promise improved performance while preserving patient privacy and minimizing data sharing risks. These innovations can support decentralized AI training on distributed data sources, enabling large-scale, privacy-conscious model recommendations for clinical integration development [85].

To facilitate clinical integration, it is essential to embed AI tools seamlessly into electronic health records (EHRs), implement intuitive user interfaces, and provide clinical staff with adequate training. Clinicians must be involved early in algorithm design and validation to ensure interpretability and real-world usability. Establishing standardized protocols for clinical testing and validation, akin to drug trials, will also be crucial for regulatory endorsement and clinician acceptance.

Technical barriers like data interoperability, system compatibility, and algorithm reliability, along with institutional challenges such as regulatory approval and workflow disruption, hinder AI integration. To overcome these, human-centered design must create intuitive, clinician-friendly tools, while dedicated training builds clinician trust and competence, both essential for smooth, effective adoption in clinical practice. Emerging AI techniques such as self-supervised and federated learning offer promising avenues for enhancing model performance while addressing data privacy and decentralization challenges. Seamless integration of AI into clinical workflows requires interoperability with electronic health records (EHRs), real-time data exchange, and user-friendly interfaces [86]. Designing AI systems that align with existing clinical pathways—such as alerts embedded in EHR dashboards—can facilitate adoption and minimize workflow disruption. Pilot studies and iterative feedback from clinicians are essential to this process.

### 7.2. Collaborative Approaches for Enhancing AI Adoption

Collaborative efforts among stakeholders, including physicians, data scientists, healthcare systems, regulatory agencies, and patients are vital to ensure AI tools are developed ethically, equitably, and effectively. Multidisciplinary research networks and consortia should foster innovation and shared learning, while policymakers must develop adaptable frameworks to support safe and equitable AI deployment [87].

## 8. Conclusions

Artificial intelligence (AI) has demonstrated significant potential in atrial fibrillation (AF) care, particularly in improving diagnostic accuracy through ECG interpretation and wearable technologies, enhancing risk prediction with advanced algorithms, and supporting therapeutic decision-making such as anticoagulation management and ablation planning. Comparative studies consistently show that AI can outperform traditional approaches in accuracy and efficiency, while predictive models integrating ECG, genetic, and biomarker data highlight opportunities for earlier and more personalized interventions.

However, despite these advances, challenges remain. Many AI tools have been validated only retrospectively, and their clinical impact depends on robust prospective trials, external validation across diverse populations, and transparent algorithm design. Ethical concerns, privacy protection, regulatory approval, and workflow integration must also be addressed to ensure safe and equitable adoption.

In conclusion, AI should not yet be considered a cornerstone of AF management, but rather a rapidly evolving set of tools with strong potential to complement clinical practice. Its successful integration will depend on rigorous validation, explainability, regulatory clarity, and seamless embedding into clinical workflows. With these conditions met, AI could meaningfully enhance personalized AF care and improve patient outcomes in the near future.

In summary, this review not only synthesizes recent advances in AI for AF diagnosis, risk prediction, and therapeutic guidance but also explicitly connects computational methodologies with their real-world clinical applications. Our focus on clinically actionable AI strategies, wearable and implantable technologies, and the ethical and regulatory frameworks necessary for adoption distinguishes this article from prior reviews. By highlighting these elements, we contribute a unique and timely perspective that informs both the development of innovative AI tools and their integration into patient-centered cardiovascular care.

## Data Availability

Not applicable.

## Abbreviations

The following abbreviations are used in this manuscript:

AF	Atrial Fibrillation
AI	Artificial Intelligence
AUC	Area Under the Receiver Operating Characteristic Curve
CHA_2_DS_2_-VA	Congestive Heart Failure, Hypertension, Age ≥75 (doubled), Diabetes Mellitus, prior Stroke or TIA (doubled), Vascular Disease, Age 65–74
CinC	Computing in Cardiology
CI	Confidence Interval
CNN	Convolutional Neural Network
DCNN	Deep Convolutional Neural Network
DL	Deep Learning
DNN	Deep Neural Network
ECG	Electrocardiogram
EHR	Electronic Health Record
FDA	Food and Drug Administration
GDPR	General Data Protection Regulation
HIPAA	Health Insurance Portability and Accountability Act
HRV	Heart Rate Variability
IHRD	Irregular Heart Rhythm Detection
LSTM	Long Short-Term Memory
ML	Machine Learning
NLP	Natural Language Processing
PPG	Photoplethysmography
PPV	Positive Predictive Value
RNN	Recurrent Neural Network
SHAP	SHapley Additive exPlanations
TIA	Transient Ischemic Attack
XAI	Explainable Artificial Intelligence
